# Automated Real-Time Identification of Medicinal Plants Species in Natural Environment Using Deep Learning Models—A Case Study from Borneo Region

**DOI:** 10.3390/plants11151952

**Published:** 2022-07-27

**Authors:** Owais A. Malik, Nazrul Ismail, Burhan R. Hussein, Umar Yahya

**Affiliations:** 1 School of Digital Science, Universiti Brunei Darussalam, Jln Tungku Link, Gadong BE1410, Brunei; 20m8540@ubd.edu.bn (N.I.); 18h8338@ubd.edu.bn (B.R.H.); 2 Department of Computer Science and Information Technology, Islamic University in Uganda, Kampala P.O. Box 7689, Uganda; umar.yahya@ieee.org

**Keywords:** deep learning, medicinal plants, species identification, computer vision, real-time system, mobile application

## Abstract

The identification of plant species is fundamental for the effective study and management of biodiversity. In a manual identification process, different characteristics of plants are measured as identification keys which are examined sequentially and adaptively to identify plant species. However, the manual process is laborious and time-consuming. Recently, technological development has called for more efficient methods to meet species’ identification requirements, such as developing digital-image-processing and pattern-recognition techniques. Despite several existing studies, there are still challenges in automating the identification of plant species accurately. This study proposed designing and developing an automated real-time plant species identification system of medicinal plants found across the Borneo region. The system is composed of a computer vision system that is used for training and testing a deep learning model, a knowledge base that acts as a dynamic database for storing plant images, together with auxiliary data, and a front-end mobile application as a user interface to the identification and feedback system. For the plant species identification task, an EfficientNet-B1-based deep learning model was adapted and trained/tested on a combined public and private plant species dataset. The proposed model achieved 87% and 84% Top-1 accuracies on a test set for the private and public datasets, respectively, which is more than a 10% accuracy improvement compared to the baseline model. During real-time system testing on the actual samples, using our mobile application, the accuracy slightly dropped to 78.5% (Top-1) and 82.6% (Top-5), which may be related to training data and testing conditions variability. A unique feature of the study is the provision of crowdsourcing feedback and geo-mapping of the species in the Borneo region, with the help of the mobile application. Nevertheless, the proposed system showed a promising direction toward real-time plant species identification system.

## 1. Introduction

Plant species’ identification is a challenging task that has a key role in effectively studying biodiversity and investigating unknown species. The manual identification of plant species is a time-consuming process and requires a lot of expertise in the field. Automated identification systems based on computer vision and machine learning techniques provide an alternative and assistive approach for this task. These systems are useful, but their accuracy varies due to the diversity of the species. A recent survey highlighted the growing application of machine learning (ML) and deep learning (DL) for plants’ identification through leaves [1]. Integrated with Mobile applications, ML and DL techniques are increasingly being applied to distinguish between diseased and healthy plants [2,3,4,5,6,7], identification and classification of herbs and medicinal plants [3,4,5,6,7,8,9,10], classification of both generic plants and specific plant species [11,12,13,14,15,16,17,18,19], identification of crop-specific diseases [20], and general identification of plants to guide field tours [21]. 

Convolutional neural networks (CNNs) and the various deep CNN models have been reported to be the most commonly used methods in the automation process of plant-classification tasks [1]. An automated diagnosis of the 10 most common tomato leaf diseases, using a mobile application, was conducted in Reference [2], using the MobileNet model of CNN, and an accuracy of up to 89% was reported. Similar to Reference [2], where a single crop (tomato) was used, researchers in Reference [7] trained and deployed Residual Neural Networks (ResNets), a deep version of CNN, in a custom-built mobile application to classify wheat disease in the wild, reporting classification accuracy of up to 96%. ResNet and Xception Networks, in combination with the YOLO object detection framework, have also been used to detect early blight tomato disease, with an accuracy of over 99% reported in Reference [3]. DL Neural Networks have also been deployed through an android-based mobile application to detect different common diseases in terrestrial plants found in the Philippines, with an accuracy of 80% [4]. B5 and B4 models of the EfficientNet DL model are reported in Reference [5], which achieved an accuracy of over 99% in the classification for 38 different diseases in various plants. However, the classification was performed in offline mode, and it was not deployed and tested over a mobile application. More recently, transfer learning has been successfully deployed in a mobile application to enable the detection of tomato leaf diseases, reporting detection accuracy of over 96%, using the EffecientNet-B0 DL model [6]. The results of these studies demonstrate the efficacy of deploying ML and DL techniques in mobile applications to detect, classify, and identify plant diseases by using plant leaves. 

Medicinal plants and herbs have continued to be used for the traditional management of various illnesses in many societies since time immemorial [22]. With advancements in artificial intelligence (AI) and different Information and Communication Technologies (ICTs), the need to automatically identify medicinal plants from the thousands of plant species can only continue to grow. Researchers in Indonesia [8] utilized local binary patterns to extract the leaf texture of 30 different medicinal plants and then applied probabilistic Neural Networks to automatically classify the herbal leaves, achieving a classification accuracy of just over 56%. In Reference [9], the researchers utilized a support vector machine (SVM) and DL Neural Networks to automatically classify 20 different herbs found in Malaysia, using a mobile application. The mobile application reported spending only 2 s for processing the input leaf image and returning the classification results, with a classification accuracy of 93%. Similarly, in Reference [10], a fusion of fuzzy local binary pattern and fuzzy color histogram, using product decision rules, was performed to enable the automatic identification of 51 medicinal plant species commonly found in Indonesia. Probabilistic Neural Networks were used to classify color histograms, reportedly achieving an accuracy of just over 74%. The promising results achieved in these studies clearly highlight the plausibility of utilizing ML and DL models in mobile applications to automate the classification of medicinal plants. 

It has been reported that there could be over 450,000 different plants, with one-third of them facing extinction [23]. The easy and automatic identification of plant species is, therefore, a crucial step toward their preservation. The scientific research community continues to make efforts toward the realization of this step. In Reference [11], the researchers built a joint-classifier by using Backpropagation Neural Networks and weighted K-NN and deployed it in an android-based mobile application to enable automatic classification of 220 plant species (angiosperms) found in China. The joint classifier reportedly achieved a classification accuracy of nearly 93%. K-NN was similarly utilized in Reference [12] to identify 32 different plant species common in Mauritius, using leaf-shape features and a color histogram; accuracy of just over 87% was reportedly achieved. In Reference [13], a custom-built android-based mobile application was able to identify a tree from 126 tree species common in the French Mediterranean area, using tree leaves, with an accuracy of up to 90%. Researchers achieved this through segmenting tree leaf images to form feature space and then used histogram intersection to predict the class. Similarly, a custom-built mobile application with a back-end classifier, using SIFT features with the Bag of Words model and SVM, was able to classify 20 different plant species common in Sri Lanka with an accuracy of 96.48% [14]. Furthermore, CNN models, including VGG19, MobileNet, and MobileNetV2, deployed in a mobile application were able to identify 33 different types of leaves common in East Hokkaido in Japan, with MobileNetV2 achieving an accuracy of over 99% [15]. In Reference [16], the researchers also deployed a CNN model in an android-based mobile application to classify natural images of leaves belonging to 129 different species crowdsourced from all over the world. Single image classification took 2.5 s, obtaining a Top-3 test error of above 60%. Similarly, in Reference [17], the researchers presented a mobile application that was able to classify plant leaves belonging to five different plant species common in India, based on leaf color and shape. To perform classification by using leaf shapes, the extracted morphological features of the leaves were categorized by using the Sobel and Otsu methods, while the color-based classification was performed by using the dominant-color method. Finally, but not least, in Reference [18], the results of a mobile application for leaf classification utilizing a CNN were presented. This study experimented with different CNN architectures’ performances in classifying 15 leaf species of plants common in California and North Carolina. Deep CNNs are reported to have achieved the highest classification accuracy of 81.6%, with the mobile application completing the classification task in 2.5 s after spending about 5.2 s loading the query leaf image in the NN classifier from the gallery. The results reported in the abovementioned works from the literature demonstrate the promising efficiency of mobile application classifiers that were developed by using the ML and DL models to facilitate the automatic identification and classification of plant species. Not only is this a crucial step toward the conservation of species risking extinction, but such mobile applications could eventually serve as field guides during tours to various plantations in the wild. In Reference [16], the researchers demonstrated how a mobile application utilizing leaf morphological features and angle code histogram was able to serve as a tour guide in a wild field with six different plant species, in the USA, with an error rate between 17% and 53%. While this error rate appears to be high, it is a promising result to begin with for a field tour in the open wild. 

The current study aimed to develop a mobile application to enable the automatic identification of medicinal plants and plants predominantly found in the Borneo region in real time. The present study builds on the strengths of promising methods established in related works from the literature, while also addressing the weaknesses (gaps) identified in the same. Most of the reviewed studies either utilized their own generated small-sized datasets for training and assessing their ML and DL models or entirely (i.e., training and testing) used some of the open existing image datasets (such as ImageCLEF, Flavia, ICL Plantae, PlantVillage, CVIP100, Flavia, Swedish, etc.), potentially resulting in overfitted and underfitted models, respectively. Additionally, many of the reviewed studies considered clean leaf images captured against a white background, a condition that is unlikely in a real-world setting in which leaf images are often captured with a non-clean background. Moreover, studies that reported excellent (over 95%) classification accuracies mainly involved classifying a single plant (e.g., tomato), thereby limiting their application to a wide range of species. Additionally, the feedback of the mobile application end-user on observed classification results was not taken into account to further enrich the classifier’s knowledge base for future classification. The development of the mobile application proposed in the current study is, therefore, a valuable addition to the existing efforts toward the preservation of the herbs and medicinal plants native to the Borneo region, a region primarily believed to be the most species-rich area in the world [24], yet with the immense threat of extinction to some of its plant species [25,26]. The current study experimented with and optimized different EffecientNet deep learning models, as transfer learning has mainly been singled-out to produce the best classification results for real-time multiclass image classification tasks [1,5]. A unique feature of the study is the provision of crowdsourcing feedback and geo-mapping of the species in the Borneo region with the help of the mobile application. This is important for the region since several local plant species (with their local names and benefits) are unknown to the experts. The system provides an adaptive learning approach where the models can be updated based on the newly collected data and people’s feedback.

Our contributions in this study are highlighted as follows:

- We have proposed a machine vision system that is capable of automating the identification of medicinal plant species in real time.
- We have developed an end-to-end computer vision system with a convolutional neural network (CNN) model to identify medicinal plant species when given an image.
- The system works in real time and can accurately identify different plant species given by simply taking a picture with a mobile camera or uploading an existing image from a device.
- The system provides a feedback mechanism and a knowledge base as a means to continuous lifelong learning of the models to produce a robust plant species identification system.

## 2. Materials and Methods 

### 2.1. Proposed System

**Figure 1:**
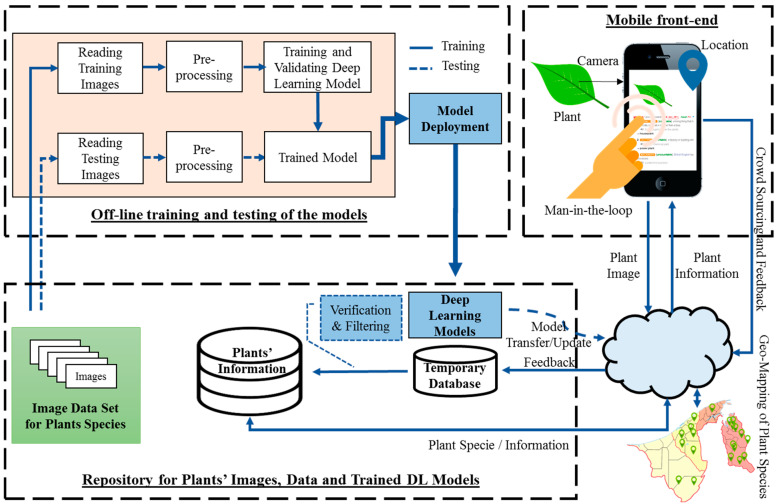
Mobile application system’s architecture.

The overall flow of the proposed system is presented in Figure 1. The system is composed of three main components: (1) a computer vision and deep-learning-based plant species classifier, (2) a knowledge base as a central repository for plant information together with auxiliary and feedback data, and (3) a mobile front-end that provides a user interface to the end-user to interact with the system and displaying of classification results. Details for each component of the system are explained in the subsequent subsections. 

### 2.2. Classification Model

To develop the real-time plant species identification system, a number of deep learning models were trained and tested on the given datasets. The details of the datasets, pre-processing steps, and training/testing of the models are described below.

#### 2.2.1. Datasets

##### PlantCLEF 2015

PlantCLEF 2015 [27] is a plant identification challenge dataset that aims to build an image-based plant identification system and evaluates methods and systems at a very large scale that adapts to real-world conditions. The dataset was constructed through a participatory community platform in 2011, consisting of thousands of collaborators. PlantCLEF 2015 consists of curated images from many different contributors, cameras, areas, periods of the year, and individual plants. More precisely, the PlantCLEF 2015 dataset comprises 113,205 pictures belonging to 41,794 observations of 1000 species of trees, herbs, and ferns living in Western European regions. Each image corresponds to one of the seven types of views in the meta-data (entire plant, fruit, leaf, flower, stem, branch, and leaf scan) and is associated with a scientific name. 

In this study, the PlantCLEF 2015 dataset was used as an auxiliary dataset to create our plant identification model to improve our classification result. We have further extracted images relevant to our application, that is, the images containing only leaf-related information (e.g., leaf, leaf-scan, and the entire plant). Thus, the extracted dataset consisted of 23,708 images. The hold-out set is built by partitioning 90% of the dataset for training and testing, while the validation data are set to be roughly 10% of the whole dataset (please see Table 1 for more detailed statistics).

##### UBD Botanical Garden Dataset

The dataset was laboriously collected from the UBD Botanical Garden, using a DSLR camera with manually annotated labels (i.e., Scientific classification of plants), together with their descriptions. The main originality of the plants in the database is that these plants are mainly native to the Borneo regions, comprising primarily medicinal plants and native plants.

**Figure 2:**
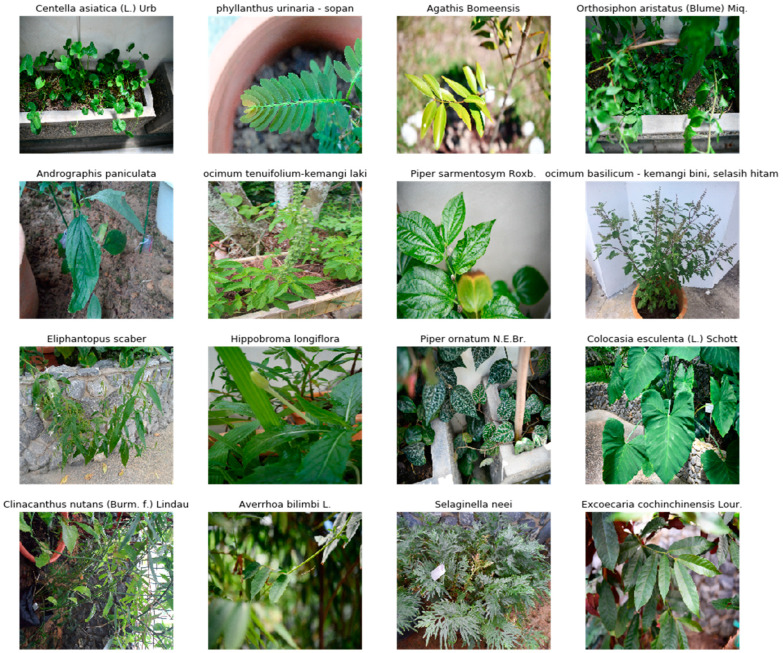
Image samples taken from UBD Botanical dataset.

The dataset consists of 106 species with a total of 2097 images collected by us (Table 2). Plants’ images were captured from different angles, and instances of plants with a variety of growth conditions were collected. Images are mostly concentrated on the leaf, with only a small fraction of the dataset containing flowers. Examples of images taken from the dataset are illustrated in Figure 2.

#### 2.2.2. Training Details

Due to the insufficient size of our dataset, we opted for transfer learning with ImageNet [28] pretrained weights. We used the EfficientNet-B1 [29] variant for deployment and added dropouts of 0.6 and a softmax layer downstream of the network. A post-training quantization of Float16 was employed by using the TensorFlow lite, resulting in 1.95 M parameters after quantization. The network was trained for 100 epochs using the Google Cloud GPU compute engine. We initially trained the model by using ImageNet weights to fine-tune to PlantCLEF 2015 dataset and later merged both datasets (i.e., PlantCLEF and UBD datasets) and performed fine-tuning of the weights in an incremental manner for the locally collected species. A total of 18,949 training Images were used from PlantCLEF and a total of 20,640 images after merging both datasets. These images were resized to 224 × 224 px with an AutoAugment [30] augmentation policy. 

AutoAugment policies are the optimal augmentation policies that improve the overall performance of models using Reinforcement Learning (RL) based on the ImageNet dataset. A policy consists of 5 sub-policies, and each sub-policy applies two image operations in sequence. Each image operation has two parameters: the probability of applying it and the magnitude of the operation. Applying the same optimal augmentation policy to different datasets while using pretrained ImageNet weights yielded an improvement in the accuracy of the models when experimented by us. Thus, we opted for ImageNet weights as our basis for transfer learning with AutoAugment policies in training our models.

**Figure 3:**
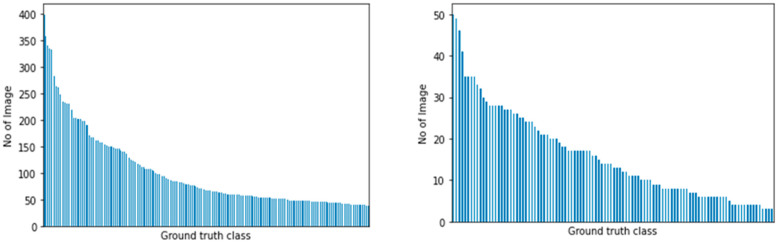
Class distribution for PlantCLEF 2015 (**left**) and UBD Botanical dataset (**right**). Ground truth class names were removed from the *x*-axis for brevity.

To counter the imbalanced distribution of samples from our datasets, we employed two cost-sensitive learning methods to train our model: computing the class weights for each class and focal loss [29]. To summarize, we trained our models by using transfer learning, using ImageNet weights with AutoAugment optimal augmentation policy and cost-sensitive learning methods applied. Figure 3 highlights the imbalance distribution for both datasets with a ratio.

##### Class Weighted Function

The classical way of training neural networks by using backpropagation involves updating the model weights with respect to errors being made by the model. This method fails when we have imbalanced training samples where examples from each class are treated the same, meaning that, for imbalanced datasets, the model is prone to performing well only for the majority class; that is, it is biased toward the majority class samples. 

The backpropagation algorithm can be updated to consider the misclassification of each class by using a cost-sensitive loss function that incorporates the error in proportion to the number of samples present in the training samples. The effect of adding this class-weighted function allows the neural network to learn the minority classes such that the model will be penalized more when misclassification of the minority class occurs. We defined the following algorithm in computing the class weights for our dataset as follows:

def class_weights($Y_{\mathrm{train}},\alpha$) #returns a dict. of class weights.
counter = dict. containing the no. of samples per class from $Y_{\mathrm{train}}$.
if α > 0:
p = max(counter.values()) * α
for class in counter.keys():
counter[class] += p
majority = max(counter.values())
return {class: $\mathrm{majority}/{\mathrm{count}}_{\mathrm{class}}\forall \mathrm{class}\;\in {\;Y}_{\mathrm{train}}$}

The α parameter represents the smoothing parameter, which balances the class weights between the majority and minority classes. It determines how much it is to penalize the model when misclassification for the minority class occurs. Setting α = 1 equates to applying equal class weightage with respect to the number of species present in the dataset and setting α > 0 equates to making the minority class weights higher. We defined α to be 0.4 for both the PlantCLEF 2015 and UBD Botanical datasets and trained by using the multiclass cross-entropy loss.

##### Focal Loss

The focal loss was introduced in Reference [29] for the object detection task, which deals with the sparse set of foreground examples present in the datasets and prevents the vast number of easy negatives from overwhelming the detector during training. The loss function was reshaped to down-weight easy examples and, thus, focused training on hard negatives by adding a modulating factor (1 − *p_t_*)*^γ^* to the cross-entropy loss, with tunable focusing parameter *γ* ≥ 0 (Equation (1)). In Reference [29], the authors experimented with *γ* ∈ [0, 5], where *γ* = 2 worked best in their experiment.

(1) $$FL\left(p_{t}\right)=-{\left(1-p_{t}\right)}^{\gamma }log\left(p_{t}\right)$$

The properties of focal loss are as follows:

- As $p_{t}$→1—meaning that the model is confident that a given sample belongs to class *t*—the modulating factor goes to 0 given γ>0, resulting in the down-weight of the loss of the easy examples during training.
- Parameter *γ*, or the focusing parameter, controls the rate at which easy examples are down-weighted. As *γ* ∈ [0, 5], *γ* = 0 is equivalent to the standard cross-entropy loss with no class weights assigned. As γ→ 5, the modulating factor grows exponentially, resulting in the increase of down-weight of easy examples.

We followed the same approach as the one described in Reference [29] for defining the γ to be 2 to both of our datasets. The experimented results for different γ values are also shown in Table 2 and Table 3.

#### 2.2.3. Hyperparameter Tuning

##### Learning Rate Finder

**Figure 4:**
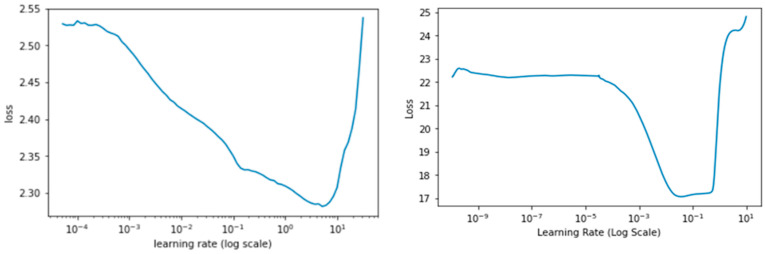
Learning rate range test with the exponential moving average for PlantCLEF 2015 (**Left**) and UBD Botanical datasets (**right**).

Following the super convergence approach for neural networks, the learning rate range test (LR range test) was performed to find the optimal LR_min_ and LR_max_ to be used in the Cyclical Learning Rate (CLR) [31,32]. The losses for each mini-batch were plotted with respect to the LR range of 10^−10^ < LR < 1, increasing exponentially on each mini-batch. The LR range test serves as a guide for how well a network performs through a range of learning rates and spot values that effectively train the network. The stopping criteria for the LR range test was set at a condition when the current loss is 4 times greater than the previous loss, as seen in Figure 4 at LR > 10^2^ regime for the PlantCLEF 2015 dataset. The “triangular” learning rate policy rule was opted for this study where learning rates oscillate in a triangular cycle from LR_min_ to LR_max_ in equal lengths of steps size. We set the LR_min_ and LR_max_ to be 10^−2^ to 10^−1^ and 10^−4^ to 10^−2^ for the PlantCLEF 2015 and UBD Botanical datasets, respectively.

### 2.3. Mobile Application Details

**Figure 5:**
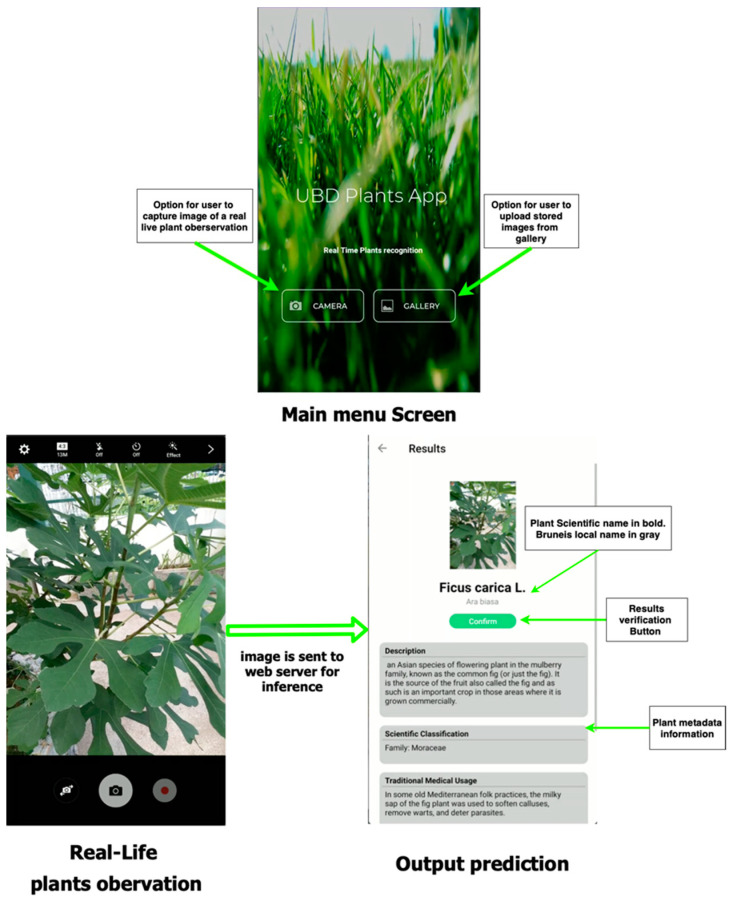
Screenshot of GUI mobile application.

We developed a mobile application dedicated to the production of our collected botanical dataset through image-based plant identification. The application currently supports Android (API level 16 and above), allowing images of a plant via gallery upload or captured by the camera to be sent to the web server to retrieve a list of predicted species made by our model. Figure 5 shows a graphical user interface of our mobile application system. Below are the details of the architecture of the mobile application system. 

#### 2.3.1. Web API and Front-End

Data exchanges between the back end (web server) and the front-end (client-side) are managed through a REST-full web API, using JSON data format and jpeg images. The list of candidate species predicted for a searched plant observation is displayed in increasing order of confidence level, and the results of only Top-3 predictions are shown to the end-user. In this study, for testing the real-time classification of the species, the Google cloud compute engine server was used as the backend (web server) to deploy the deep learning model, and the custom-developed mobile applications worked as the front-end. 

#### 2.3.2. Species Prediction

The predictions made by the classifier are based on the scientific or botanical names of the plant. Metadata information (i.e., family, genus classification, plant usage, etc.) is further extracted from a database via a web server. If a candidate species has a weak confidence level by the model, the observation may not correspond to one species inside our database (rejection class). As a result, the list of returned Top-3 predicted results is not displayed, and the end-users are allowed to suggest the name of the observation.

#### 2.3.3. User Response Information Storage

Each top-k label returned by the model may not be relevant or accurate; as a result, we have implemented a verification mechanism that allows the end-users to verify the returned results and store the response. Information stored includes one or several plant images tagged with provenance data (device, author, date, etc.). Each observation is associated with a geo-location and one or more determinations, i.e., possible species names proposed by the author him/herself, and/or by other annotators, and/or by automated classifiers. Observations are stored within a NoSQL database called MongoDB. The observations are later verified by domain experts and can be used to improve the model performance as a continuous lifelong learning process for plant species identification.

### 2.4. Knowledge Base

A knowledge base (KB) is a centralized repository for information related to a particular field or domain. In this study, a KB was created in order to manage the information related to the plant species’ profiles and other ancillary data. The KB contains different types of information, including raw and processed image data, domain knowledge, learning models (trained intelligent methods), geo-location of images transferred by users for classification, feedback data, etc. The designed KB is not a static collection of information; instead, it acts as a dynamic resource that has the capacity to learn and evolve with time when new plant images are presented, and new classification/labels are added to the system. Thus, as an integral component of the plant species classification system, this KB has been used to optimize the collection, organization, and retrieval of relevant information for plants. The internal implementation of the KB is a hybrid storage model consisting of a database, image files, and classification models.

## 3. Experiments and Results

The trained classification models were evaluated in two stages: Initially, the models were tested offline on a hold-out test set from both datasets. In the next phase, the models were deployed to the mobile application for real-time species identification, wherein their performance was assessed. 

We selected accuracy, sensitivity (true-negative rate), and specificity (true-positive rate) as evaluation metrics, computed as the average for all class labels present in the target dataset. Specificity presents the proportion of the predicted negative classes which were correctly predicted. On the other hand, sensitivity presents a proportion of the true-positive classes which were correctly identified. 

The equations for these metrics are given below:

(2) $$Specificity=\frac{\sum_{i=1}^{k}TP_{i}}{\sum_{i=1}^{k}(TP_{i}+FN_{i})}$$

(3) $$Sensitivity=\frac{\sum_{i=1}^{k}TN_{i}}{\sum_{i=1}^{k}(TN_{i}+FP_{i})}$$

(4) $$Accuracy=\frac{\sum_{i=1}^{k}\frac{TP_{i}+TN_{i}}{TP_{i}+TN_{i}+FP_{i}+FN_{i}}}{k}$$

where *k* represents total class labels; and *TN*, *FN*, *FP*, and *TP* are the numbers of the true-positive, true-negative, false-positive, and false-negative predictions for the considered class, respectively.

### 3.1. Offline Testing of the Classification Models (for Both Datasets)

**Figure 6:**
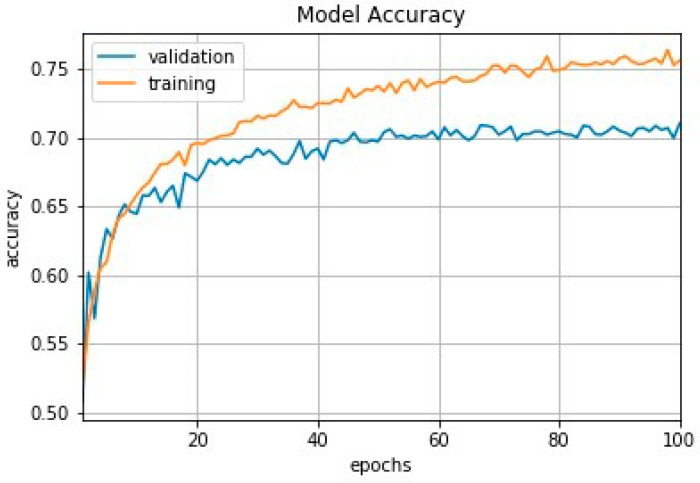
Training and validation loss curves for the deep learning model.

We trained a baseline model by applying the cost-sensitive learning method to our highly imbalanced dataset with standard cross-entropy loss and applied the same training techniques as outlined in Section 2.2.2. The training and validation loss curves for the model are shown in Figure 6. We performed an experiment for a total of three runs and reported its average evaluation metrics, as shown in Table 3 and Table 4 for both datasets. The results shown in these tables are based on 2380 test samples for PlantCLEF 2015 and 2537 samples when merged (PlantCLEF and UBD Botanical dataset), respectively.

The baseline model showed roughly 80% in Top-5 accuracy. However, it performs poorly in classifying the true-positive and true-negative samples as visible from the low sensitivity and specificity rate. The baseline model was found to be biased toward the majority classes; hence, it had a high accuracy rate (80%) and a low rate of sensitivity or recall (43.2%). Both cost-sensitive learning methods, including the class weighted and focal loss, showed an improvement in the classification performance for the imbalanced dataset, as indicated by the significant increase in the sensitivity and specificity values. With the class weighted function applied, the model is being penalized more and able to make a correct prediction with an improvement of sensitivity rate from 43% to 79%, while using focal loss has the effect of having the model recognize classes that do not correspond to its labels. We later performed an evaluation on the test samples on the merged dataset and ran a total of three runs. The results shown in Table 4 are based on a total of 251 species with 2537 images. 

The baseline model appeared to show no improvement, albeit adding more samples from our collected dataset. This is mainly due to the imbalance in the nature of the dataset, as shown in the distribution graph in Figure 3. After applying the cost-sensitive learning methods and adding more external samples, a slight incremental improvement in performance was found. In the next section, we outlined the test performed on real-life plant observation and reported its performance.

### 3.2. Mobile Application Testing

Based on the species identification performance shown in Table 3 and Table 4, we selected two models for testing where both are trained on the merged dataset with the highest sensitivity for each of the respective cost-learning methods, i.e., focal loss with *γ* = 2.0 and class weighted function with α = 0.4. The real-life plants’ observation test samples used for this study are the 121 species which are found at the UBD Botanical Garden.

Due to the lack of variability in our dataset, both models’ performance deteriorates upon real-time testing, as shown in Table 5. The accuracy of using focal loss is comparable to offline testing, with an average of 80% accuracy for both Top-1 and Top-5 accuracy. However, the sensitivity drops for both methods’ classes weighted function and focal loss from 80% to 66.1% and 75.2%, respectively. The resultant of this performance may have been caused by the failure of the model to generalize the dynamic change of state of the plants (i.e., growth cycle), which can be solved when more samples are present in the dataset.

**Figure 7:**
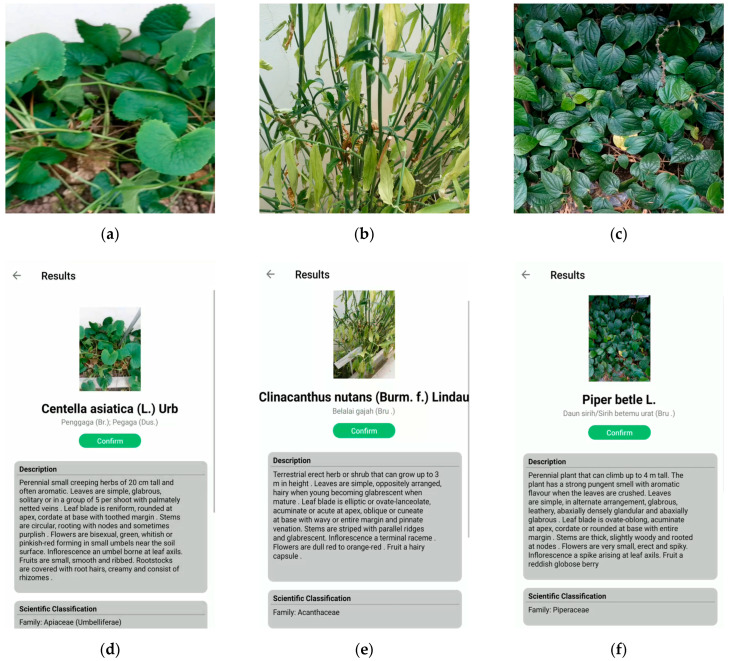
Examples of real-time classification of some species, using the mobile application: (**a**) *Centella asiatica* (L.) Urb; (**b**) *Clinacanthus nutans* (Burm. f.) Lindau; (**c**) *Piper betle* L.; (**d**) real-time classification of *Centella asiatica* (L.) Urb; (**e**) real-time classification of *Clinacanthus nutans* (Burm. f.) Lindau; (**f**) real-time classification of *Piper betle* L.

**Figure 8:**
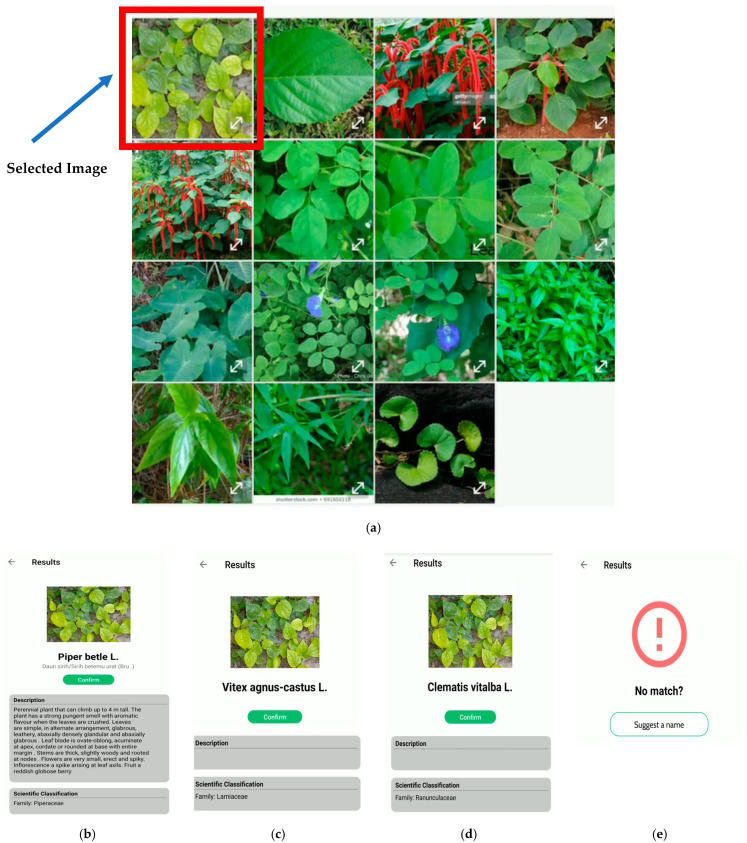
Examples of offline classification of a plant by using the mobile application and feedback option for correction/suggestion for the plant name. (**a**) Images from the gallery for offline testing: (**b**) first matching, (**c**) second matching, (**d**) third matching, and (**e**) feedback option.

Some examples of the real-time testing of the developed mobile application are shown in Figure 7. In the real-time classification system, the image taken through the mobile camera is uploaded to the cloud, and the classification results are sent back to the mobile device. The corresponding information, including the plant’s scientific name, description, medical usage, precautions, local family name, common name, and origin, is retrieved from the online database and displayed to the user. Since the system also facilitates the geo-mapping of the species, the longitude and latitude information, where the user took the image, can also be saved in the knowledge base. Moreover, a confirmation option is provided for the users to give feedback to improve the system further, and the domain expert verifies this at the end. This option helps us continuously improve the classification model for plant species identification. Figure 8 depicts an example of the system when used in the offline mode. It also shows a scenario where a plant image can be classified into multiple species with different probability values. The class with the highest probability value is shown first, while the rest are shown in decreasing order of probability values. Similar to the real-time system, a feedback mechanism is provided to report the misclassification of the plant species.

In contrast to several previous promising studies in which either open-access datasets [2,3,5] or localized primary datasets [4,20] were exclusively utilized, the deep learning models trained and tested in the current study used over 25,000 images, consisting of both secondary open-access datasets, as well as a primary dataset of images collected from a local botanical garden in Brunei with medicinal plants native to Borneo region. Therefore, this dataset diversity adds to the reproducibility confidence of the obtained results under real-time conditions and the likely generalizability of the trained models. Additionally, the average real-time in-the-wild classification accuracy of 80% for both Top-1 and Top-5 accuracies obtained in the current study is lower than the classification accuracies of 88.4% and 99.9% previously reported in References [2,3], respectively; it should be noted, however, that the latter studies were dealing with diseased leaf detection for only one type of crop (tomatoes) in contrast to the present study, with over 250 medicinal plants species. Furthermore, as a way of building on previous related works [2,4,6,17,20,21] that aimed to train and deploy deep learning plants identification models on mobile applications, the current work has introduced a new feature by enabling confirmation and, thus, verification of the classification results by the mobile application user, as shown in Figure 5. This new feature introduced in this work aids the continuous improvement of the cloud-hosted knowledge base, particularly by domain experts and native users of medicinal plants, thereby facilitating incremental learning of the deployed deep learning model.

## 4. Conclusions and Future Work

In this study, a deep-learning-based system was proposed to perform a real-time species identification of medicinal plants found in the Borneo region. The proposed system addressed some of the key challenges when training deep learning models, such as small training samples with long-tile class imbalance distribution of the species data. Techniques such as class weighting and the use of focal loss function were applied to improve the learning process of the model. The results showed that the proposed system could significantly improve the performance of the deep learning model by more than 10% accuracy compared to the baseline model. However, performance accuracy was slightly dropped when the system was tested on the actual samples by using the developed mobile application in real time. In the future, we intend to further improve the system’s performance by improving the sample collection of the training data. Furthermore, to make the system more useful, we intend to increase the number of species, as the Borneo region is a high species diversity spot. 

## Figures and Tables

**Table 1:** Number of images extracted from PlantCLEF 2015 dataset.

	Total	Leaf	Leaf-Scan	Entire Plant
Train	18,949	6122	9522	3305
Test	2380	767	1190	423
Validation	2379	781	1134	464

**Table 2:** Numbers of images for UBD Botanical dataset.

Train	1691
Test	157
Validation	249
Total	2097

**Table 3:** Performance evaluation on PlantCLEF 2015 test set.

	Top-1 Acc. (%)	Top-5 Acc. (%)	Sensitivity (%)	Specificity (%)
Baseline	73.5	79.4	43.2	53.5
Class weighted (α=0.2)	81.9	87.4	61.5	64.6
Class weighted (α=0.4)	83.2	92.4	79.5	77.6
Focal Loss (γ=2.0)	83.8	92.2	76.5	74.6
Focal Loss (γ=5.0)	84.0	89.4	76.5	74.6

**Table 4:** Performance evaluation on UBD Botanical + PlantCLEF test set.

	Top-1 Accuracy (%)	Top-5 Accuracy (%)	Sensitivity (%)	Specificity (%)
Baseline	63.4	72.4	43.2	53.5
Class weighted (α=0.2)	83.5	87.4	61.5	64.6
Class weighted (α=0.4)	85.5	92.4	73.5	77.6
Focal Loss (γ=2.0)	83.5	92.4	71.5	77.6
Focal Loss (γ=5.0)	87.5	86.4	70.5	74.6

**Table 5:** Performance evaluation on real-life plants observation at UBD Botanical Garden.

	Top-1 Accuracy (%)	Top-5 Accuracy (%)	Sensitivity (%)	Specificity (%)
Focal Loss (γ=2.0)	78.5	82.6	75.2	77.7
Class weighted (a=0.4)	62.8	70.2	66.1	68.6

## Data Availability

The data that support the findings of this study are available upon request.
